# Crossed cerebellar diaschisis after stroke detected noninvasively by arterial spin-labeling MR imaging

**DOI:** 10.1186/s12868-020-00595-z

**Published:** 2020-11-20

**Authors:** Juan Wang, Li-Jun Pan, Bin Zhou, Jin-Yan Zu, Yi-Xu Zhao, Yang Li, Wan-Qiu Zhu, Lei Li, Jian-Rong Xu, Zeng-Ai Chen

**Affiliations:** 1 Department of Radiology, Renji Hospital, School of Medicine, Shanghai Jiao Tong University, 1630 Dongfang Rd, Shanghai, 200127 China; 2 Department of Radiology, Renji Hospital South Campus, School of Medicine, Shanghai Jiao Tong University, 2000 Jiangyue Rd, Shanghai, 201112 China

**Keywords:** Crossed cerebellar diaschisis, Arterial spin-labeling, Single-photon emission CT, Stroke

## Abstract

**Background:**

As a noninvasive perfusion-weighted MRI technique, arterial spin-labeling (ASL) was becoming increasingly used to evaluate cerebral hemodynamics in many studies. The relation between ASL-MRI and crossed cerebellar diaschisis (CCD) was rarely discussed. In this study, the aim of our study was to assess the performance of ASL-MRI in the detection of crossed cerebellar diaschisis after stroke in compared with single-photon emission CT (SPECT).

**Results:**

17 of 51(33.3%) patients revealed CCD phenomenon by the SPECT method. In CCD-positive group, CBF_*ASL*_ of ipsilateral cerebellar were significantly increased compared with contralateral cerebellar (*p* < 0.0001) while no significant differences (*p* = 0.063, > 0.001) in the CCD-negative group. Positive correlation was detected between admission National institute of health stroke scale (NIHSS) and asymmetry index of SPECT (AI_*SPECT*_) (*r* = 0.351, *p* = 0.011), AI_*ASL*_ (*r* = 0.372, *p* = 0.007); infract volume and AI_*SPECT*_ (*r* = 0.443, *p* = 0.001), AI_*ASL*_ (r = 0.426, *p* = 0.002). Significant correlation was also found between cerebral blood flow of SPECT (CBF_*SPECT*_) and CBF_*ASL*_, AI_*SPECT*_ and AI_*ASL*_ (*r* = 0.204, *p* = 0.04; *r* = 0.467, *p* = 0.001, respectively). Furthermore, the area under the receiver operating characteristic (ROC) curve value of AI_*ASL*_ was 0.829.

**Conclusions:**

CBF derived from ASL-MRI could be valuable for assessment of CCD in supratentorial stroke patients. Additionally, CCD was significantly associated with larger ischemic volume and higher initial NIHSS score.

## Background

Crossed cerebellar diaschisis (CCD), defined as a depression of metabolism and blood flow in the cerebellar hemisphere contralateral to a supratentorial infarct, was first described in 1981 [1]. CCD was consistently reported in several disorders that result in neuronal damage or depletion,, such as status epilepticus [2], supratentorial infarction [3], glioma [4] and lymphoma [5]. Previous studies found that CCD is a secondary neuronal depression which attributed to disruption of the cortico-ponto-cerebellar pathways with consecutive cerebellar functional inactivation [6]. The previous studies of CCD were frequently reported on single-photon emission CT (SPECT) [7] and positron-emission tomography (PET) [8]. However, the use of radioactive tracers and the high price limit their repeatability and application for healthy volunteers. Thus, it’s a pressing need to find a noninvasive and widely available imaging technology in clinical for detecting CCD.

With the development of MR techniques, several studies have been carried out to detect CCD by diffusion-tensor imaging (DTI) and perfusion-weighted MR imaging (PW-MRI). A study found that DTI could visualize the cortco-ponto-cerebellar pathway in CCD with chronic stroke which rarely demonstrated by conventional MR images [9]. Dynamic susceptibility contrast perfusion weighted-imaging (DSC-PWI) was found to be able to identify CCD by various parameters but with a reduced sensitivity compared with PET/SPECT [10]. In addition the sinus artifacts and susceptibility artifacts attributed to the skull in the posterior fossa may be another important limitation in detection of CCD. Our previous study also demonstrated that the fast diffusion coefficient derived from the intravoxel incoherent motion (IVIM) could be valuable for assessing CCD in supratentorial stroke [3].

As a new non-contrast-enhanced perfusion imaging method, arterial spin labeling (ASL) has widely been applied for quantitative regional CBF measurement. Briefly, ASL takes endogenous arterial water as a diffusible tracer, which enables us to estimate brain perfusion frequently over a long-term follow-up period [11]. So far, several studies have reported the relation between ASL-MRI and CCD. Though Chen S, et al. observed that ASL was useful to detect CCD after stroke because of the consistency with PET/SPECT series [12], while the limitation was a lack of head-to-head comparison with PET/SPECT imaging. Furthermore, the asymmetry indices (AIs) of CCD obtained by using ASL were significantly correlated with those obtained by using SPECT in the previous study by Kang KM, et al. [13].Therefore, the purpose of our study was to evaluate the feasibility of ASL-MRI in CCD detection after supratentorial stroke and compare ASL with SPECT imaging in the assessment of CCD.

### Results

As shown in Table 1, among all 51 patients, 17 (33.3%) exhibited CCD by SPECT method. Patients were divided into CCD-positive and CCD-negative groups using SPECT as reference. The CCD-positive and CCD-negative groups did not differ in age and sex (*p* = 0.78, *p* = 0.286, respectively). There was no significant difference in mean duration from stroke onset to ASL imaging between the CCD-positive and CCD-negative groups (5.82 ± 3.41 days, 5.10 ± 2.79 days, respectively; *p* = 0.596); the mean time interval from stroke onset to SPECT maps was 5.27 ± 2.73 days and 4.93 ± 4.30 days, respectively (*p* = 0.131). After clinical treatment, the discharge National institute of health stroke scale (NIHSS) score was significantly decreased than admission score in the CCD-negative group (*p* < 0.0001), whereas no significant difference was found between admission and discharge NIHSS scores in the CCD-positive group (*p* = 0.272). Higher AI_*SPECT*_ and AI_*ASL*_ value was investigated in the CCD-positive group compared to CCD-negative group respectively, and there was no significant difference between AI_*SPECT*_ and AI_*ASL*_ (*p* = 0.484).

**Table 1:** Demographic characteristics of CCD (+) and CCD (−) subjects

Characteristics	ALL (51)	CCD + (17)	CCD − (34)	*p* value^a^	*p* value
Age (year)	62.16 ± 12.16	62.5 ± 12.04	61.47 ± 13.10	0.78	/
Male sex-(%)	35 (68.6%)	10 (58.8%)	25 (73.5%)	0.286	/
Infarct volumne (mm^3^)	10559 ± 20431	23953 ± 30124	3861 ± 6242	0.006*	/
Stroke to SPECT imaging (d)	5.04 ± 3.83	5.27 ± 2.73	4.93 ± 4.30	0.131	/
Stroke to ASL imaging (d)	5.34 ± 3.00	5.82 ± 3.41	5.10 ± 2.79	0.596	/
Admission NIHSS	3.12 ± 2.16	4.29 ± 2.71	2.52 ± 1.61	0.021*	0.272^b^
Discharge NIHSS	2.53 ± 2.19	4.00 ± 2.52	1.79 ± 1.64	0.002*	< 0.0001^c, #^
AI_SPECT_	0.07 ± 0.09	0.1 ± 0.08	0.02 ± 0.05	< 0.0001*	/
AI_*ASL*_	0.09 ± 0.12	0.19 ± 0.10	0.03 ± 0.10	< 0.0001*	0.484^d^

Values are mean ± standard deviation or number of patients

CCD, crossed cerebellar diaschisis; NIHSS, Initial National Institute of Health Stroke Scale; AI, asymmetry index; CBF, cerebral blood flow; SPECT, single-photon emission CT; ASL, arterial spin-labeling MR imaging

^*, #^ The significant level was defined as *P* < 0.05

^a^Comparison of parameters in CCD ( +) and CCD (−)

^b^ Comparison of NIHSS score at admission and discharge in CCD (+) subjects

^c^ Comparison of NIHSS score at admission and discharge in CCD (−) subjects

^d^comparison of AI_SPECT_ and AI_ASL_

**Fig. 1:**
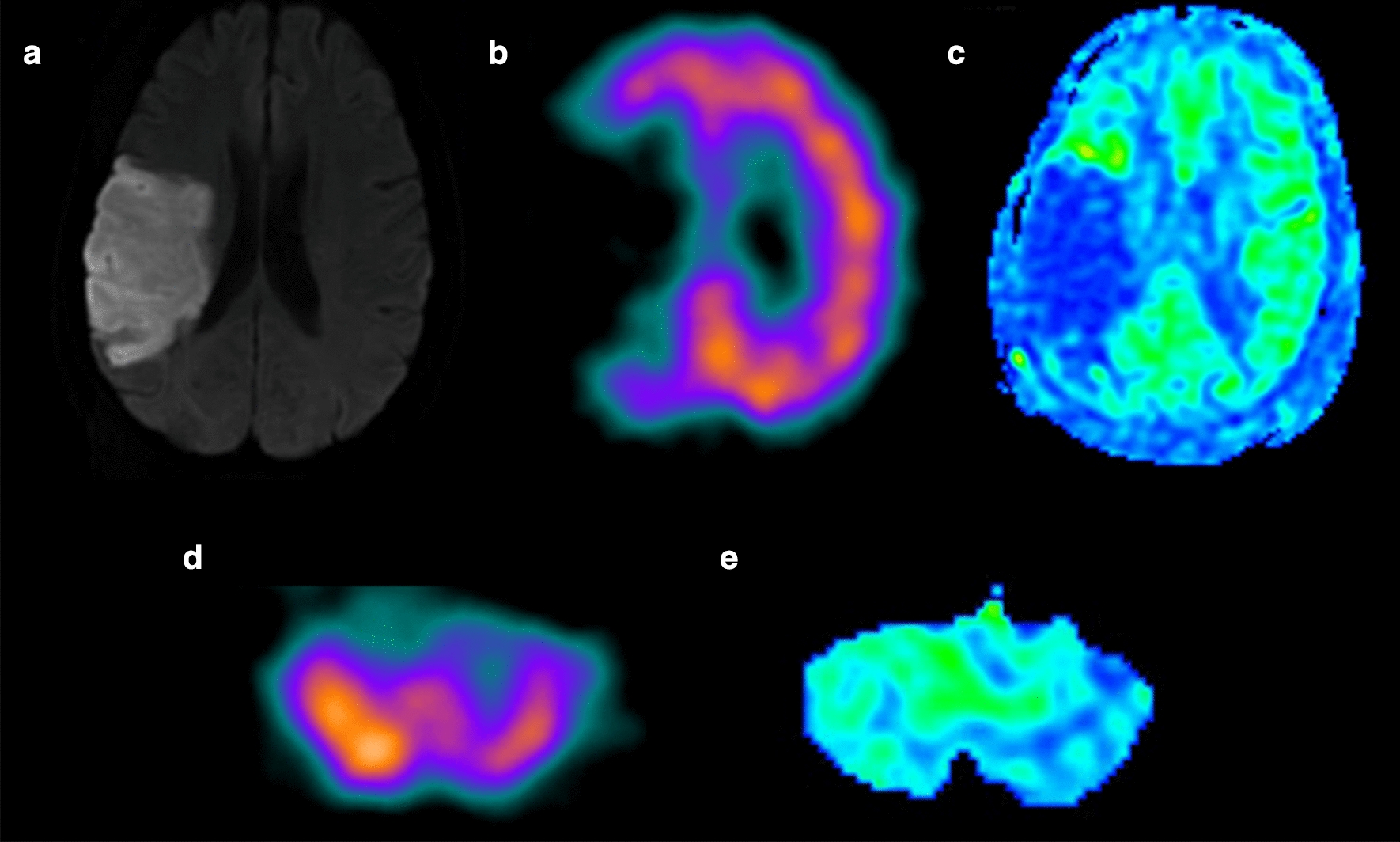
A 62‑year‑old man with a history of sudden onset left-sided weakness for 7 days. **a** Hyperintensity stroke lesion can be recognized in the right temporal lobe on diffusion weighted image. **b** SPECT and **c** ASL maps showing hypoperfusion in the same lesion. **d** SPECT and **e** ASL images showing hypoperfusion in the contralateral cerebellum hemisphere

The median CBF_*ASL*_ of ipsilateral cerebellum (39.86 ± 9.43 ml/100 g/min) was significantly increased than that of contralateral cerebellum (33.34 ± 9.87 ml/100 g/min, *p* < 0.0001) in the CCD-positive group, while no significant difference was detected in the CCD-negative group (p = 0.063). Furthermore the similar phenomenon was also observed in CBF_*SPECT*_ (Table 2). Figure 1 shows the images of DWI (A), SPECT (B,D) and ASL(C,E) of a representative patients with supratentorial stoke in right parietal for 7 days.

**Table 2:** Comparison of CBF_*SPECT*_, CBF_*ASL*_ in bilateral cerebellum

Group	Parameters	Ipsilateral cerebellum	Contralateral cerebellum	*p* value
CCD +	CBF_*SPECT*_ (ml/100 g/min)	83.35 ± 24.04	66.49 ± 18.83	< 0.0001*
	CBF_*ASL*_ (ml/100 g/min)	39.86 ± 9.43	33.34 ± 9.87	< 0.0001*
CCD−	CBF_*SPECT*_ (ml/100 g/min)	75.18 ± 27.73	73.51 ± 25.9	0.007*
	CBF_*ASL*_ (ml/100 g/min)	42.36 ± 10.16	40.99 ± 10.09	0.063

CBF, cerebral blood flow; SPECT, single-photon emission CT; ASL, arterial spin-labeling MR imaging

Values are mean ± standard deviation. * The significant level was defined as *P* < 0.05

**Fig. 2:**
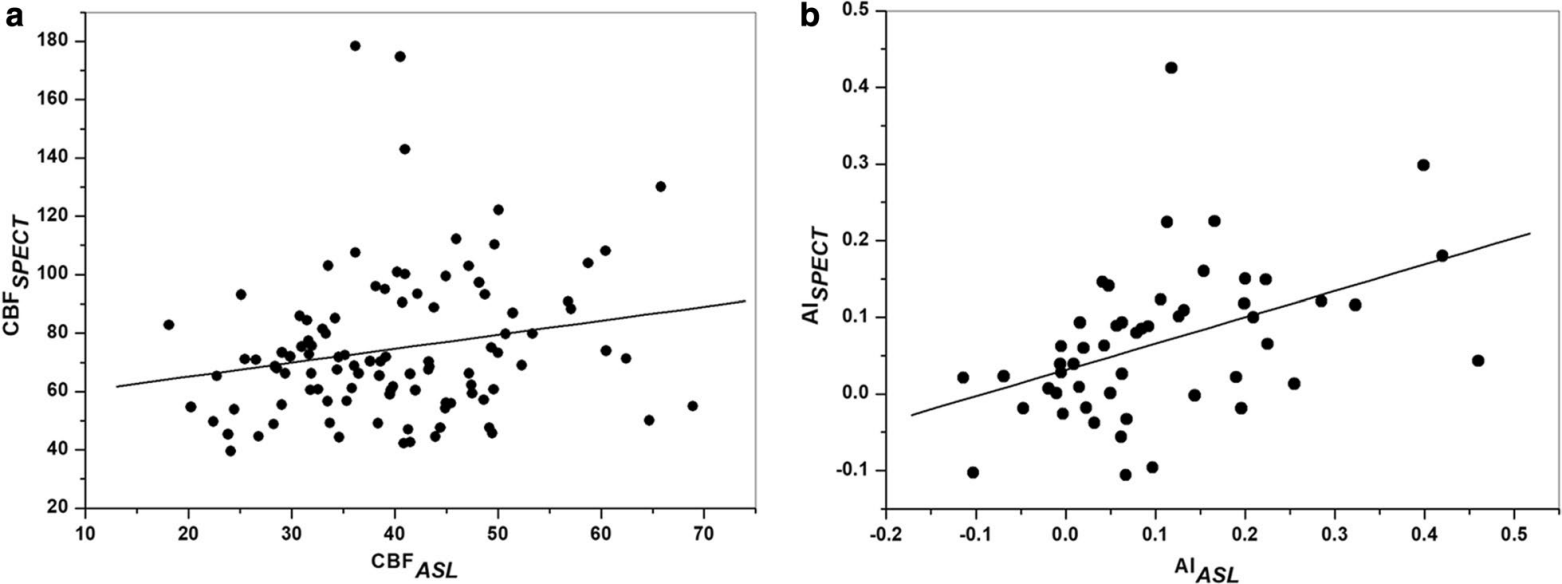
The scatter plot for relationships between CBF_*ASL*_ and CBF_*SPECT*_ (**a**) (r = 0.201, p = 0.043) and AI_*ASL*_ and AI_*SPECT*_ (**b**) (r = 0.467, p = 0.001)

**Fig. 3:**
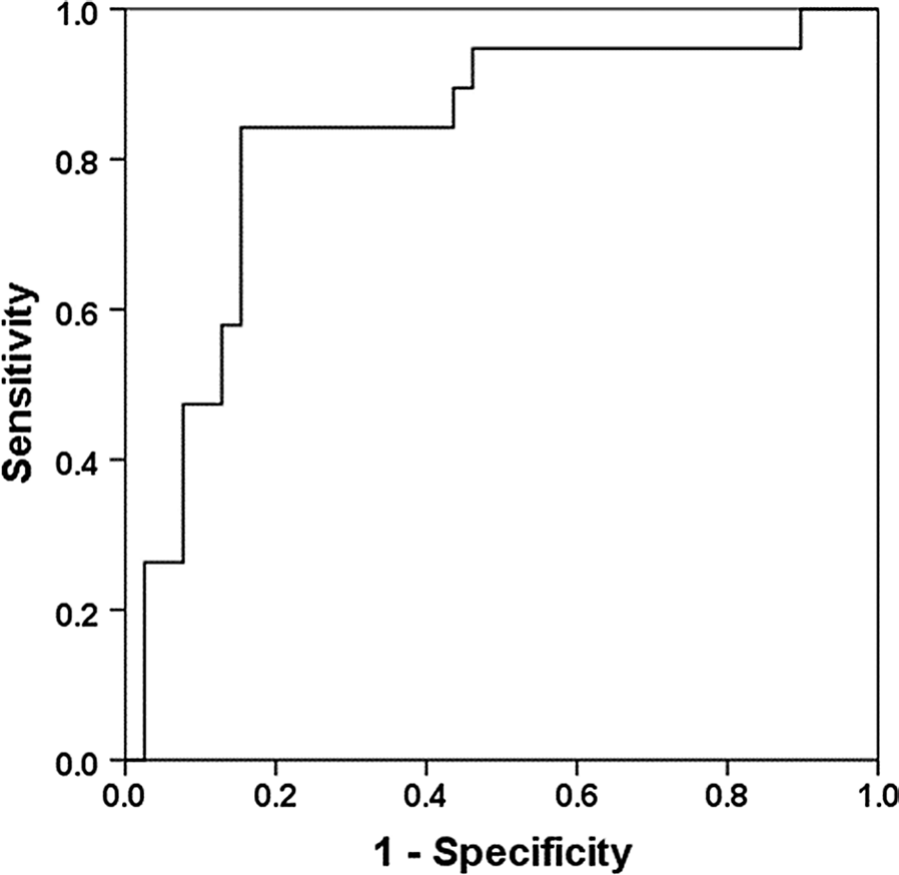
Receiver operating characteristic (ROC) curves for AI_*ASL*_ vs AI_*SPECT*_

The results of Pearson’s correlation analysis are presented in Table 3. There were positive correlations between admission NIHSS and AI_*SPECT*_ (*r* = 0.351, *p* = 0.011) and AI_*ASL*_ (*r* = 0.372, *p* = 0.007); infract volume and AI_*SPECT*_ (*r* = 0.443, *p* = 0.001), and AI_*ASL*_ (r = 0.426, *p* = 0.002). Additionally, significant correlation was also found between CBF_*SPECT*_ and CBF_*ASL*_ (*r* = 0.204, *p* = 0.04), AI_*SPECT*_ and AI_*ASL*_(*r* = 0.467, *p* = 0.001) (Fig. 2a and b). The area under the ROC curve value of AI_*ASL*_ for CCD diagnosis was 0.829 (Fig. 3). The sensitivity, specificity, positive likelihood ratio (+ LR) and negative likelihood ratio (− LR) values of AI_*ASL*_ in CCD diagnosis were 84.2%, 84.6%, 3.81 and 0.33 respectively, at the optimal cut-off value of 10%.

**Table 3:** The results of Person’s correlation between various parameters

Parameters	*r* value	*p* value
AI_*SPECT*_ and admission NIHSS	0.351	0.011*
AI_*ASL*_ and admission NIHSS	0.372	0.007*
AI_*SPECT*_ and infract volume	0.443	0.001*
AI_*ASL*_ and infract volume	0.426	0.002*
CBF_*ASL*_ and CBF_SPECT_	0.201	0.043*
AI_*ASL*_ and AI_SPECT_	0.467	0.001*

AI, asymmetry index; CBF, cerebral blood flow; SPECT, single-photon emission CT; ASL, arterial spin-labeling MR imaging; NIHSS, Initial National Institute of Health Stroke Scale

^*^The significant level was defined as *P* < 0.05

## Discussion

As PET and SPECT is currently considered as the standard for the detection of CCD, our study firstly displayed a comparison of ASL-MRI at 3.0 T with SPECT imaging in CCD measurement directly. According to this study, first of all, the CBF_*ASL*_ of ipsilateral cerebellar (40.40 ± 9.81 ml/100 g/min) was significant higher than that of contralateral cerebellar (34.15 ± 11.23 ml/100 g/min) in CCD-positive group. The major finding was meeting the imaging criteria for CCD defined as reduced baseline cerebellar CBF contralateral to supratentorial infarct. In additions, there was a positive correlation between CBF_*ASL*_ and CBF_*SPECT*_, AI_*ASL*_ and AI_*SPECT*_, respectively, which implying that ASL might reflect the CBF alteration in post-stroke CCD patients and be a novel imaging method for CCD detection.

As a new contrast-free and radiation-free imaging method, ASL permits a quantitative measurement of cerebral perfusion and repetitive acquisitions with independent labeling [14, 15]. ASL is widely used as a noninvasive and repeatable brain perfusion imaging technology in many diseases such as brain tumors, cerebrovascular diseases and acute stroke. Noguchi et al. [16] indicated that perfusion imaging by ASL-MRI could be used to recognize the condition of brain perfusion in the clinical evaluation of moyamoya disease (MMD). rCBF derived from pseudo-continuous arterial spin labeling (PCASL) could offer a convenient and noninvasive imaging method of perfusion in the human brain in comparison with SPECT [17]. 3D-ASL also has proved to be a useful method for detecting CCD in branch atheromatous disease [18]. Our present findings supported these conclusions and observed ASL against SPECT imaging in CCD detection. There was a positive correlation between CBF_*ASL*_ and CBF_*SPECT*_, AI_*ASL*_ and AI_*SPECT*_ respectively, which is consistent with the previous study [13]. With SPECT as reference, the area under the ROC curve was 0.829, indicating that CBF_*ASL*_ maps may manifest CCD as well as SPECT.

The lower detection rate of CCD (33.3%) in our series compared with the PET (85% ~ 89%) [19, 20] or SPECT (46%) [21] literature may be related to multiple factors: but the major may be the various imaging principles. A highly diffusible tracer, O-water used in PET which may explain why PET has a high sensitivity to depict subtle hemodynamic alterations. Conversely, PW-MRI has lower incidence of CCD compared with PET because of the methodical limitations in the posterior fossa such as sinus srtifacts and so on [20]. Besides that, the median time interval from onset to acquire images and the differences in patient selection may influence the occurrence of CCD. Acute CCD is a functional process and may be rapidly reversible when the excitatory input to the cerebellum returns (e.g., in the case of supratentorial reperfusion in patients with stroke) [6, 22]. Persistence of CCD is an irreversible process which leading to transneuronal degeneration and usually resulting in atrophy of the affected cerebellar hemisphere [23].

In addition, infarction size and severity might also account for the various occurrence of CCD. Previously, a PET study on CCD detecting reported that the volume of supratentorial infract played a significant role in CCD development [8]. This suggestion is compatible with our results in which the infract volume showed a favorable correlation with AI_*ASL*_ and AI_*SPECT*_. Shinohara et al. proved that the asymmetry index of the contralateral cerebellar hemisphere was significantly correlated with NIHSS score by using 3D-ASL which implied that NIHSS score may play an important role in the degree of neurologic severity [18]. In our study, the significant positive relation was observed between admission NIHSS and AI_*ASL*_, admission NIHSS and AI_*SPECT*_, respectively. Furthermore, the NIHSS score reduced significantly in CCD-negative cases at discharge, but showed no significant difference between admission and discharge in CCD-positive cases. These results demonstrated that the NIHSS score and infarct volume were closely related to CCD-positive and patients with CCD may have worse outcome than those without CCD in subacute stroke.

## Conclusions

Crossed cerebellar diaschisis can be diagnosed with ASL-MRI by decrease CBF_*ASL*_ in the contralateral cerebellum compared with that in the ipsilesional side. We found that CCD was more prevalent in patients with larger ischemic volumes and higher initial NIHSS. However, our study has limitations. First, this is a retrospective study of comparative MRI and SPECT imaging available but with a modest sample size. Second, perfusion changes after stroke between the imaging procedures cannot be ruled out, the interval between MRI and SPECT in this study is relatively long. Third, ROI-based methods in our study may be inaccurate for technique comparisons. In our further study, we will expand sample size and patients will receive comparative imaging within a narrow time frame. As a rapid, noninvasive, and quantitative technique, ASL would be a novel and valuable method for evaluating CCD after subacute hemispheric stroke.

## Methods

### Patients

From August 2013 to December 2014, a total of 51 patients (35 males and 16 females; mean age 62.16 ± 12.16 years, age range 34–84 years) diagnosed with subacute ischemic stroke who underwent 3.0-T MRI and SPECT were included in this study. All patients were admitted to hospital suffering from symptoms of acute hemispheric stroke and were subsequently diagnosed with subacute ischemic stroke in the middle cerebral artery territory. The definition of acute and subacute stroke according to temporal evolution was based on the literature by Kim et al.: acute (6–24 h), and subacute (24 h to approximately 2 weeks) [24]. The mean duration from stroke onset to MR imaging in was 5.34 ± 3.00 days and the mean time interval from stroke onset to SPECT was 5.04 ± 3.83 days. The time interval between SPECT and ASL-MRI acquisition is 0.92 ± 0.78 days. The National Institute of Health Stroke Scale (NIHSS) was used to evaluate the neurological and functional status at the time of the admission and at discharge (14 days later).

Patients in the following situations were excluded from our study: (1) with infarct in the brain stem, cerebellum or bilateral supratentorial infarct; (2) with history of intracranial tumor, head trauma, subarachnoid hemorrhage, arteriovenous malformation, or brain surgery; (3) with abnormalities in the posterior fossa on T1,T2, and diffusion-weighted MR images; (4) with magnetic resonance angiography (MRA) showing angiopathy of vertebral basilar artery and the major branches; and (5) with incomplete coverage of the posterior fossa in SPECT or MRI imaging.

### Magnetic resonance imaging

Magnetic resonance imaging was performed on a 3.0-T magnetic resonance scanner (HDxt; General Electric Medical Systems, Waukesha, WI, USA) using a standard 8-channelphase array head coil. Sequences obtained were conventional T_1_WI, T_2_WI, diffusion weighted imaging (DWI), MRA, and ASL. DWI was performed with the following parameters: *b*-value = 1000 s/mm^2^, repetition time (TR) = 6000 ms, field of view = 24 cm, matrix = 128 × 128, slice thickness = 5 mm, skip = 1.5 mm, slices = 20, number of excitations (NEX) = 2. Gradients were encoded in 3 directions to create isotropic DWI apparent diffusion coefficient (ADC) maps. Pseudo-continuous ASL perfusion images were collected using 3D fast spin echo acquisition with background suppression, with post labeling delay of 1500 ms. TR = 4601 ms, TE = 10.5 ms, field of View = 24 cm, matrix = 128 × 128, NEX = 3, slice thickness = 4 mm, and slices = 38.

### SPECT imaging

SPECT was performed in all patients in a silent, dimly lit room with eyes open and ears unplugged. 20–25 min after intravenous injection of 925–1110 MBq (25-30 mCi) technetium-99methylcysteinatedimer (99mTc-ECD, HAT CO. LTD Shanghai, China), acquisition was done on a dual-headed rotating scintillation gamma camera (Infinia Hawkeye 4, GE Healthcare) with the patient supine, headrest attached, smallest permissible radius of rotation, 128 × 128 matrix, 360°, 120 projections, 25 s per view for a total 64 views by using a low-energy high-resolution parallel hole collimator. A 20% window centered at a 140 keV photo peak for Tc-99 m was used. Raw data were smoothed with Butterworth filtered.

### Imaging data processing

ASL-MRI images were transferred to a standard workstation (GE Advantage Workstation 4.5) for post-processing. Image analysis was performed with the ASL module integrated in Functool software. Circular regions of interest in ASL-CBF images, measuring 30 mm indiameter, were placed in the cerebellar hemispheres ipsilateral (I) and contralateral (C) to the hemispheric stroke. We calculated asymmetry indices (AI) for the cerebellar hemisphere as following formula:

$${\text{AI}}_{{SPECT}} = \left( {CBF_{{\text{I}}} - CBF_{{\text{C}}} } \right)/\left( {CBF_{{\text{I}}} + CBF_{{\text{C}}} } \right) \times {\text{2}}00\%.$$

The presence of CCD was defined as AI_*SPECT*_ > 10% [25]. The same method was used to obtain AI_*ASL*_ according to:

$${\text{AI}}_{{ASL}} = \left( {CBF_{{\text{I}}} - CBF_{{\text{C}}} } \right)/\left( {CBF_{{\text{I}}} + CBF_{C} } \right) \times {\text{2}}00\%.$$

All region of interests (ROIs) were placed to avoid the major vessels and cerebellar vermis. The ischemic volume was obtained from DWI imaging, which was calculated by using the following formula: Ischemic volume = length*width*height/2. To evaluate the inter-reader reproducibility, these ROIs and ischemic volume values were measured by two experienced neuroradiologists both with 5 years of experiences.

## Data analysis

All the date were expressed as mean ± standard deviation (SD). Statistical analysis was performed by using SPSS 20.0 (SPSS, Chicago, IL, USA). Patients were divided into CCD-positive group and CCD-negative group according to AI_*SPECT*_. To compare the CCD-positive and CCD-negative groups, descriptive data were analyzed using the Mann–Whitney U test or independent-sample *t* test for non-categorical data as appropriate; meanwhile the Fisher exact test was applied for categorical variables. Paired *t* tests were used for comparison of the CBF_*ASL*_ and CBF_*SPECT*_ between ipsilateral and contralateral cerebellar in CCD-positive and CCD-negative groups respectively. For exploring the relationship among various parameters, Pearson’s or Spearman’s correlation was used. Bland–Altman plots were generated to display the spread of data and the limits of agreement. Receiver operating characteristic (ROC) curve was used to investigate the accuracy of ASL-MRI to detect CCD. Statistical significance was defined as *p* < 0.05 or *p* < 0.001(two-tailed) respectively.

## Data Availability

All data used and analyzed during the current study available from the corresponding author on reasonable request.

## Abbreviations

ASL: Arterial spin-labeling
SPECT: Single-photon emission CT
CCD: Crossed cerebellar diaschisis
NIHSS: National institute of health stroke scale
ROC: Receiver operating characteristic
CBF: Cerebral blood flow
PET: Positron-emission tomography
DTI: Diffusion-tensor imaging
MRA: Magnetic resonance angiography
DWI: Diffusion weighted imaging
ADC: Apparent diffusion coefficient
ROI: Region of interest
AI: Asymmetry index
